# Advancements and applications of loop-mediated isothermal amplification technology: a comprehensive overview

**DOI:** 10.3389/fmicb.2024.1406632

**Published:** 2024-07-17

**Authors:** Nan Yang, Han Zhang, Xiu Han, Zhifeng Liu, Yan Lu

**Affiliations:** ^1^Department of Gastroenterology, Children’s Hospital of Nanjing Medical University, Nanjing, China; ^2^Institute of Translational Medicine, Medical College, Yangzhou University, Yangzhou, China

**Keywords:** loop-mediated isothermal amplification, monitoring methods, DNA polymerase, primer design, molecular diagnosis, technical improvement

## Abstract

Loop-mediated isothermal amplification (LAMP) is a novel method for nucleic acid detection known for its isothermal properties, high efficiency, sensitivity, and specificity. LAMP employs 4 to 6 primers targeting 6 to 8 regions of the desired sequence, allowing for amplification at temperatures between 60 and 65°C and the production of up to 10^9^ copies within a single hour. The product can be monitored by various methods such as turbidimetry, fluorometry, and colorimetry. However, it faces limitations such as the risk of non-specific amplification, challenges in primer design, unsuitability for short gene sequences, and difficulty in multiplexing. Recent advancements in polymerase and primer design have enhanced the speed and convenience of the LAMP reaction. Additionally, integrating LAMP with technologies like rolling circle amplification (RCA), recombinase polymerase amplification (RPA), and CRISPR-Cas systems has enhanced its efficiency. The combination of LAMP with various biosensors has enabled real-time analysis, broadening its application in point-of-care testing (POCT). Microfluidic technology has further facilitated the automation and miniaturization of LAMP assays, allowing for the simultaneous detection of multiple targets and preventing contamination. This review highlights advancements in LAMP, focusing on primer design, polymerase engineering, and its integration with other technologies. Continuous improvements and integration of LAMP with complementary technologies have significantly enhanced its diagnostic capabilities, making it a robust tool for rapid, sensitive, and specific nucleic acid detection with promising implications for healthcare, agriculture, and environmental monitoring.

## Graphical abstract

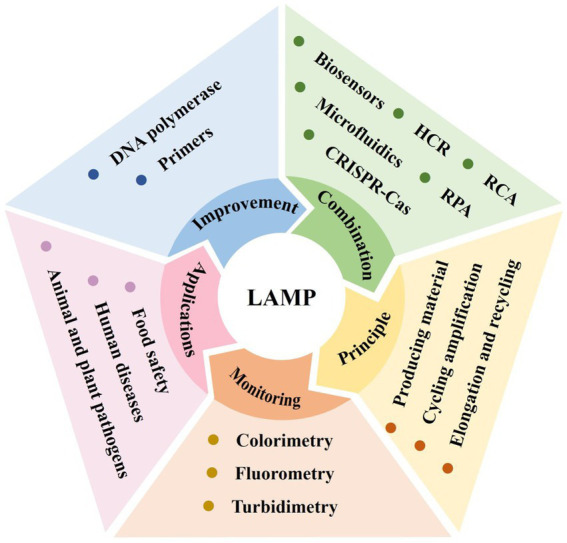

## 1 Introduction

Loop-mediated isothermal amplification (LAMP) is a molecular diagnostic technology introduced by Notomi et al. (2000), enabling rapid nucleic acid amplification at a constant temperature. Unlike conventional PCR, which requires temperature cycling, LAMP achieves continuous rapid amplification under isothermal conditions using strand-displacing DNA polymerase and a set of four specific primers targeting six regions of the target gene. This design allows for high sensitivity, enabling amplification from a very small number of copies. The ultimate results of LAMP can be detected through turbidimetry, fluorometry, and colorimetry, making it more suitable for on-site testing (Park, 2022).

LAMP’s isothermal nature eliminates the need for expensive thermal cyclers, making the technique more accessible, especially in resource-limited settings. Its high sensitivity and rapid amplification capabilities make it particularly useful for detecting low-abundance nucleic acids in clinical, agricultural, and environmental samples (Wei et al., 2022; Jiang et al., 2023; Søndreli et al., 2023). Furthermore, the method’s ability to provide visual readouts simplifies result interpretation, facilitating its use in field diagnostics and point-of-care testing (POCT).

Despite the robustness, LAMP has a few limitations. The presence of multiple primers in LAMP assays increases the risk of non-specific amplification, leading to false positive results (Kim et al., 2023). Furthermore, LAMP is not suitable for short gene sequences (Ding et al., 2019), and designing multiple primers poses challenges for researchers (Hu et al., 2016). Achieving multiplexing in a single tube is another significant limitation (Lin et al., 2015). To address these challenges, significant advancements have been made in LAMP technology. Researchers have focused on improving primer design and DNA polymerase modification to enhance the specificity and efficiency of the reaction. Additionally, the integration of LAMP with innovative molecular techniques and biosensors has expanded its capabilities. These enhancements have made LAMP a more versatile and powerful tool for nucleic acid detection (Soroka et al., 2021).

This paper aims to provide a comprehensive review of LAMP technology, including its basic principles, recent advancements, and diverse applications. We will discuss the latest improvements in LAMP assay design and explore how these innovations have broadened its use in various fields. By highlighting the strengths and addressing the limitations of LAMP, we aim to demonstrate its potential as a robust tool for rapid, sensitive, and specific nucleic acid detection, with significant implications for healthcare, agriculture, and environmental monitoring.

## 2 The principle of LAMP

**Figure 1:**
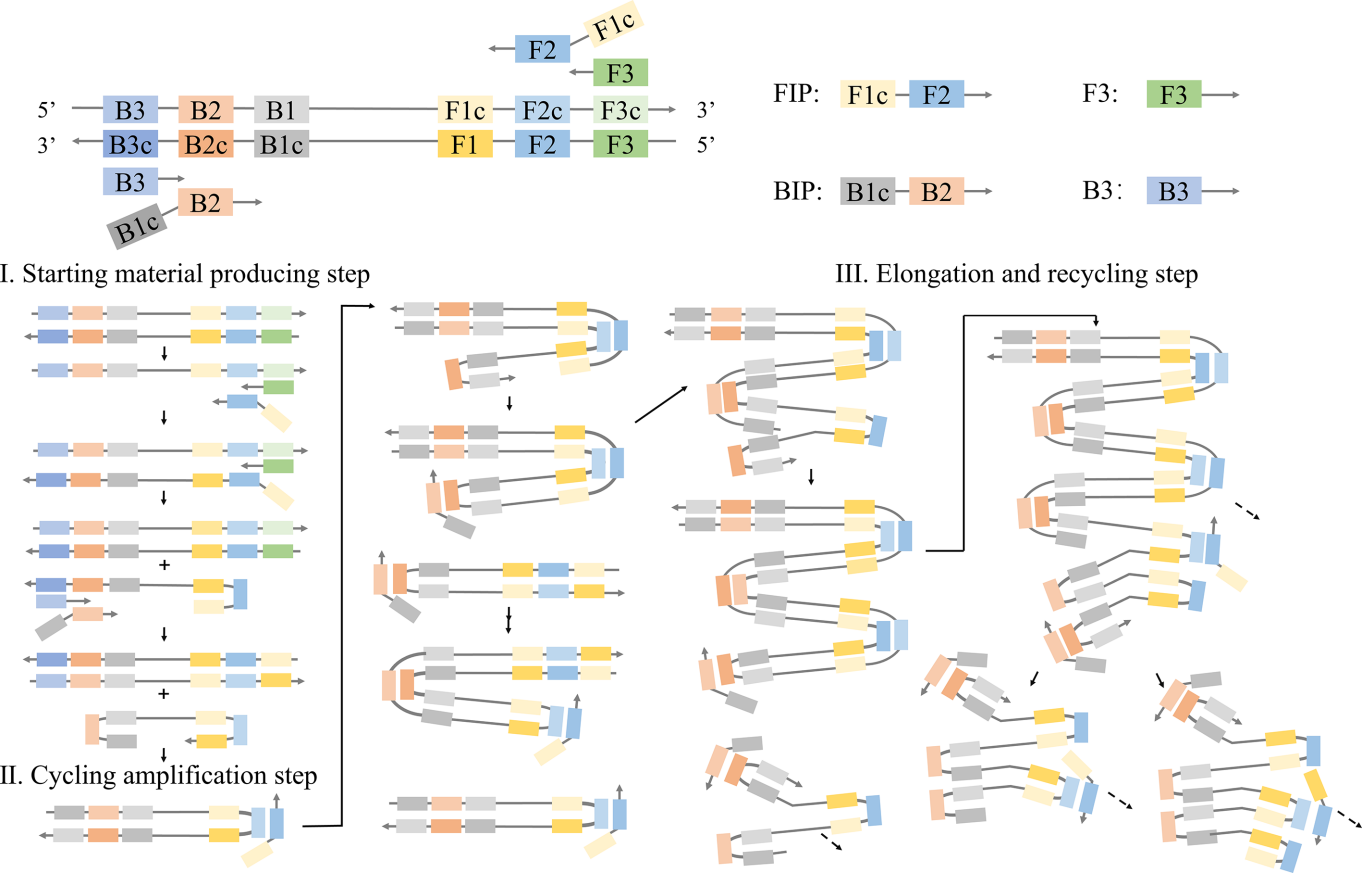
Schematic representation of the mechanism of LAMP.

In the LAMP method (Notomi et al., 2000; Figure 1), primers are designed to target six fragments of the desired gene. In this reaction, pairs of internal primers (FIP and BIP) and external primers (F3 and B3) are used. Internal primers guide the formation of stem-loop structures, while external primers facilitate strand displacement. Specifically, FIP (FIc + F2) and F3 primers hybridize with template strands F2c and F3c, respectively. Upon the addition of DNA polymerase, FIP, and F3 are elongated and formed stem-loop structures. The stem-loop structure is facilitated by the presence of complementary F1c and F1 sequences at the 5′ end of the FIP-linked strand. This FIP-linked strand then acts as a template for BIP (B1c + B2) and B3 primers, ultimately resulting in the generation of dumbbell-shaped DNA with stem-loop structures at each end. This dumbbell-shaped DNA structure then serves as the foundational template for the following LAMP process, which generates a stem-loop DNA chain that is double the length of the initial stem, utilizing itself as a template. Through this template, the synthesis and strand replacement processes persist, resulting in the formation of DNA strands containing numerous repetitive target sequences and stem-loop structures.

## 3 Monitoring methods

### 3.1 Turbidimetry

In the LAMP process, a significant amount of pyrophosphate ion by-product is generated, resulting in the formation of a white precipitate of magnesium pyrophosphate within the reaction mixture. The alteration in turbidity, indicative of precipitation formation, correlates directly with the quantity of synthesized DNA, enabling real-time monitoring of the LAMP reaction through the utilization of real-time turbidimeters (Mori et al., 2001). Inagaki et al. developed a rapid detection technique utilizing the amplification refractory mutation system-LAMP method for the identification of the macrolide resistance gene in *Mycobacterium avium*. This approach enables the rapid assessment of drug resistance through real-time turbidimetry within a short period (Inagaki et al., 2024).

### 3.2 Fluorometry

Fluorescent dyes, such as SYTO-9, SYTO-13, SYTO-82, SYBR Green I, SYBR Gold, and EvaGreen, which selectively bind to double-strand DNA (dsDNA), facilitate visible color change upon formation of the dye-dsDNA complex. Consequently, these dyes can be utilized to monitor the products of the LAMP reaction (Oscorbin et al., 2016). A real-time LAMP method was developed for the detection of *Enterocytozoon hepatopenaei* using the DNA binding dye SYTO-16, which demonstrated a limit of detection (LOD) of 10 copies/μL (Ma et al., 2019). Lai et al. conducted a study in which they developed a real-time fluorescence LAMP assay for the detection of zoonotic malaria *Plasmodium parasites*. This assay utilized SYTO-9 as the nucleic acid intercalating dye. The amplification process was successfully completed within a 60-min timeframe, with an LOD ranging from 5 × 10^9^ to 5 × 10^10^ copies/μL (Lai et al., 2024).

### 3.3 Colorimetry

Unlike turbidimetry and fluorometry, which require specialized equipment such as turbidimeters and fluorometers, colorimetry can be performed with the naked eye, making it more suitable for on-site detection. During LAMP amplification, protons (H^+^) are generated. As the pH decreases, the pH indicator xylenol orange changes from purple to yellow, which enables the visual detection of analytes (Tanner et al., 2015). Another colorimetric method relies on the reduction of Mg^2+^. Calcein, for instance, is deprived of Mn^2+^ by accumulating pyrophosphate ions, resulting in a visible color change from orange to yellow when it binds to Mg^2+^ (Tomita et al., 2008). Similarly, the colors of eriochrome black T and hydroxy naphthol blue change from violet to sky blue as the Mg^2+^ level decreases (Goto et al., 2009; Neshani et al., 2023). Neshani et al. (2023) reported a closed-tube LAMP for the visual detection of *Mycobacterium tuberculosis*, comparing the effectiveness of calcein, eriochrome black T, and hydroxy naphthol blue as indicators. Their results showed that hydroxy naphthol blue-, calcein-, and eriochrome black T-LAMP could respectively detect 100 fg, 1 pg, and 1 pg DNA/reaction (Neshani et al., 2023).

## 4 Improvement of LAMP

### 4.1 DNA polymerase

Bst DNA polymerase (Bst DNA pol) (Aliotta et al., 1996), originating from *Bacillus stearothermophilus*, exhibits 5–3’ DNA polymerase activity and specific 5–3′ exonuclease activity on double-stranded DNA while lacking 3–5′ exonuclease activity. The polymerases utilized in isothermal amplification technologies typically consist of large fragments of Bst DNA pol without exonuclease activity. The strand displacement capability of Bst DNA pol is sufficiently robust to facilitate the separation of DNA strands at a constant temperature. The thermal stability and polymerase activity of Bst DNA pol are major considerations for isothermal amplification techniques. Advancements in biotechnology have led to the engineering of variants of Bst DNA pol for use in isothermal amplification. In 2012, New England BioLabs obtained a patent for Bst 2.0, which has demonstrated superior performance in polymerization speed, thermal stability, salt tolerance, and dUTP tolerance compared to Bst DNA pol (Oscorbin and Filipenko, 2023). The introduction of Bst 2.0 WarmStart has enabled the initiation of reactions at room temperature to prevent nonspecific amplification and enhance reaction efficiency (Shirshikov and Bespyatykh, 2022). Furthermore, New England BioLabs has developed Bst 3.0 by fusing Bst 2.0 with an additional DNA binding domain, resulting in a variant with reverse transcriptase activity. Bst 3.0 demonstrates remarkable capabilities in amplification even under challenging conditions with amplification inhibitors and impure samples. Bst 3.0 is also able to amplify RNA templates. Ghaith et al. utilized Bst 3.0 in LAMP for the rapid detection of the Foot-and-Mouth Disease Virus (FMDV) within 15 min (Ghaith and Abu Ghazaleh, 2021).

In addition to Bst DNA pol and its derivatives, OmniAmp polymerase (OmniAmp pol) is also suitable for LAMP. Derived from viral metagenomes found in the hot springs of Yellowstone National Park, OmniAmp pol exhibits inherent reverse transcriptase activity, thermal stability, and efficient strand displacement activity. Chander et al. (2014) discovered that OmniAmp pol outperforms Bst DNA pol in terms of speed, particularly when amplifying highly diluted templates. OmniAmp pol can efficiently amplify DNA or RNA templates in just 30 min, which is 20% shorter than traditional LAMP methods. Ye et al. (2021) utilized enzymatic engineering to combine Bst DNA pol with the FEN1 enzyme, resulting in the creation of the recombinant FEN1-Bst DNA pol. This novel polymerase possesses the ability to perform DNA synthesis, strand displacement, and cleavage functions, enabling the detection of as few as 10 copies/μL of rotavirus, *Chlamydia trachomatis*, and the SARS-CoV-2 N gene in LAMP assays.

### 4.2 Primer of LAMP

#### 4.2.1 Primers for higher speed and higher sensitivity

**Figure 2:**
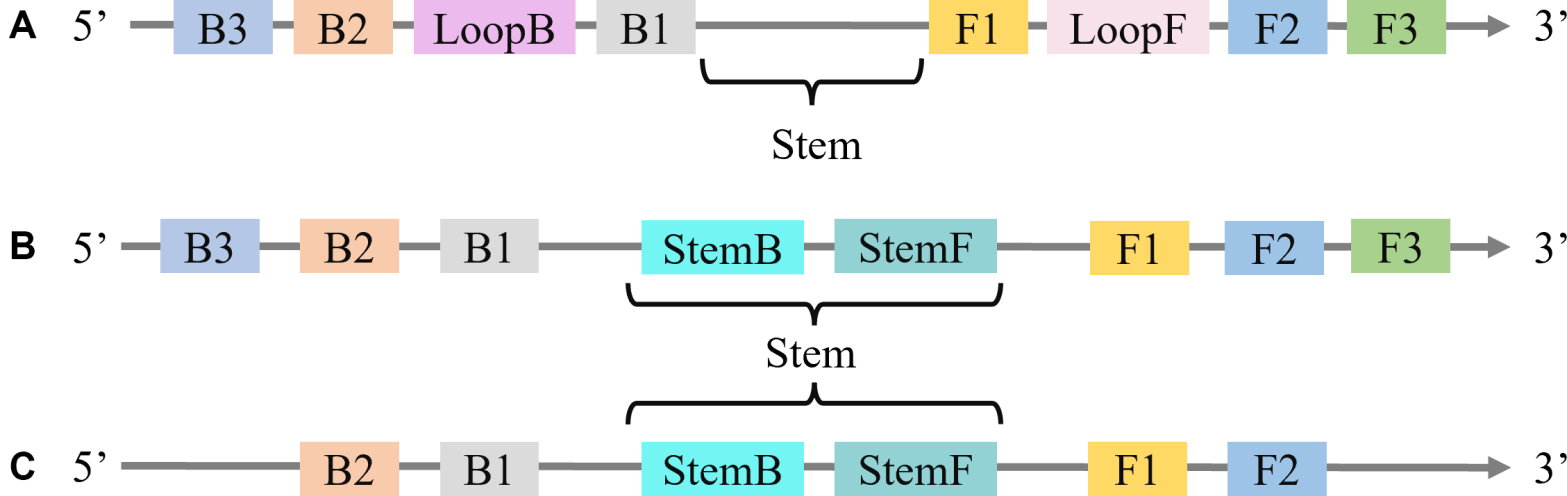
**(A)** The “Stem” in LAMP with Loop primers. **(B)** Location of Stem primers in LAMP. **(C)** LAMP with Stem primers but without “Displacement primers”.

Nagamine et al. (2002) proposed the incorporation of loop primers (LoopF and LoopB) in the design of the LAMP technique, resulting in a 30 to 50% acceleration of the original process. However, the precise binding of the loop primer to the stem-loop structure presents a significant challenge in primer design. Gandelman et al. (2011) introduced the STEM-LAMP method which utilized stem primers (as they target the “Stem” in the LAMP amplicon) in place of loop primers (Figure 2). This method demonstrated high efficacy in detecting *Clostridium difficile*, *Listeria monocytogenes*, and human immunodeficiency virus.

Compared to the design limitations of loop primers, stem primers can be used in both forward and reverse orientation, which provides more flexibility. Their research revealed that stem primers exhibit similar reaction speed and sensitivity to loop primers. Furthermore, stem primers can also be combined with loop primers (Figure 2A), to eliminate displacement primers (Figure 2C). Specifically, Njiru et al. (2017) utilized stem primers in LAMP for the detection of *Trypanosoma gambiense*, achieving a 100-fold increase in sensitivity compared to traditional LAMP methods utilizing loop primers. Additionally, Mwendwa et al. (2017) employed stem primers to detect *Entamoeba histolytica* based on the 18S small subunit ribosomal RNA gene, achieving a detection time of 11 min with a sensitivity of 30 pg/mL.

#### 4.2.2 Primers for lower reaction temperature

The optimum temperature range of LAMP is between 60 and 65°C. However, Cai et al. (2018) have introduced a variant of LAMP known as phosphorothioated primers LAMP through the modification of primers F1c and B1c with phosphorothioate. This modification enables amplification at 40°C while maintaining a sensitivity level similar to that of the original LAMP method.

#### 4.2.3 Primers for amplifying short gene sequences

**Figure 3:**
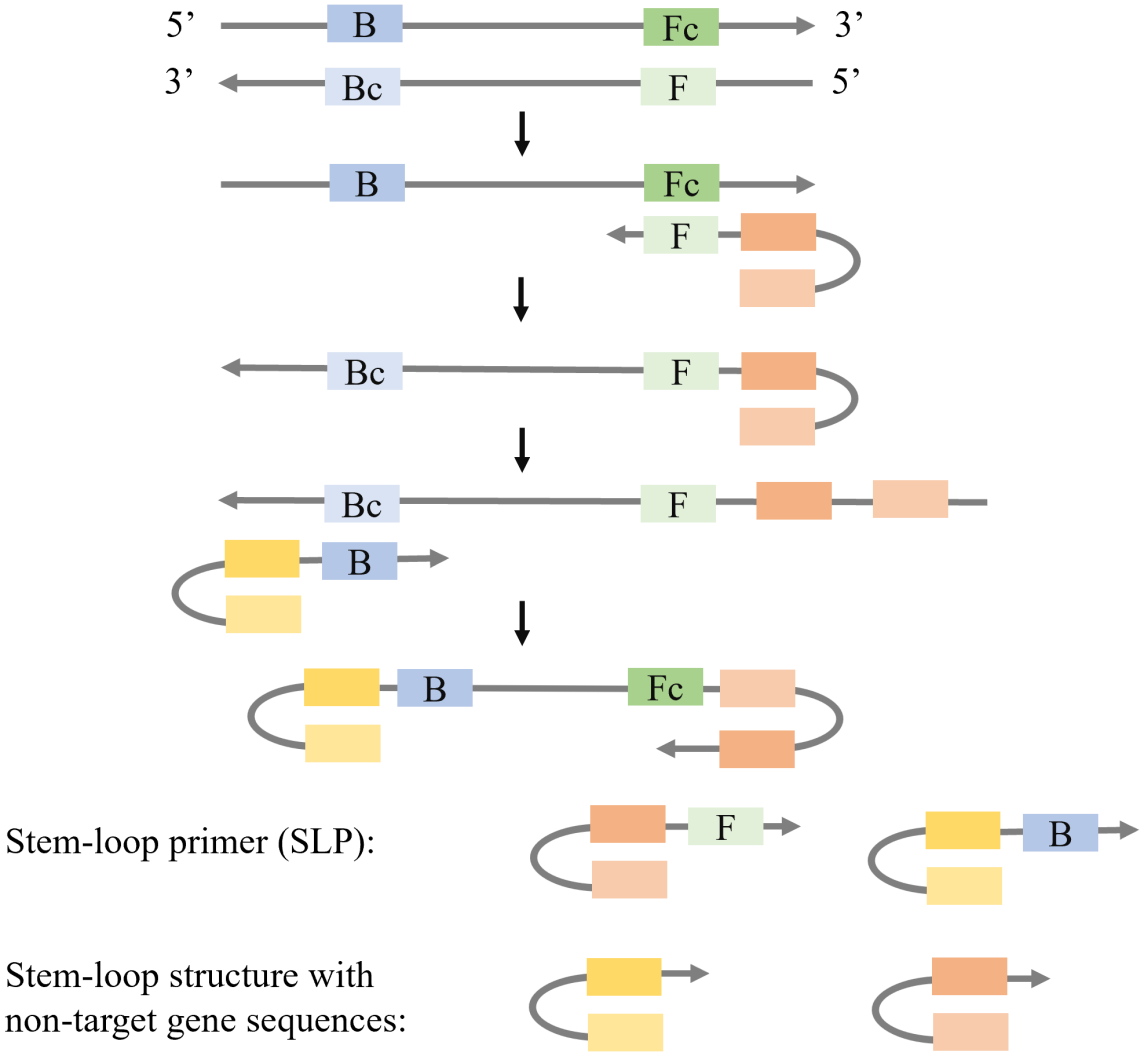
Schematic presentation of the mechanism of LAMP with Stem-loop primers (SLPs).

Due to the utilization of multiple pairs of primers in LAMP, the formation of primer dimers and non-specific amplifications is prone to occur. Short gene sequences require specific primers to eliminate primer-dimer formation leading to the use of stem-loop primers (SLPs) as solution (Figure 3). The SLPs are characterized by a stem-loop structure with non-target gene sequences at the 5′ end and linear structures at the 3′ end that are complementary and capable of hybridizing with the target gene. The forward SLP identifies the target gene and generates a complementary strand with a stem-loop structure, while the backward SLP utilizes this complementary strand as a template to produce dumb-bell DNA with stem-loops at both ends, initiating subsequent LAMP reactions. Ding et al. (2019) introduced a novel approach known as single enzyme-based stem-loop and linear primers co-mediated exponential amplification of short gene sequences (SLIMP), which demonstrated the ability to utilize an 80 nt hepatitis B virus gene as the template and detect target genes at concentrations as low as 1 aM within a 60-min time. Additionally, Luo et al. (2021) introduced Stem-loop-primer assisted isothermal amplification (SPA) as a method for the detection of Human Papilloma virus types 16, 18, 52, and 58 in cervical samples by using a target gene of 40 to 80 bp in length.

**Figure 4:**
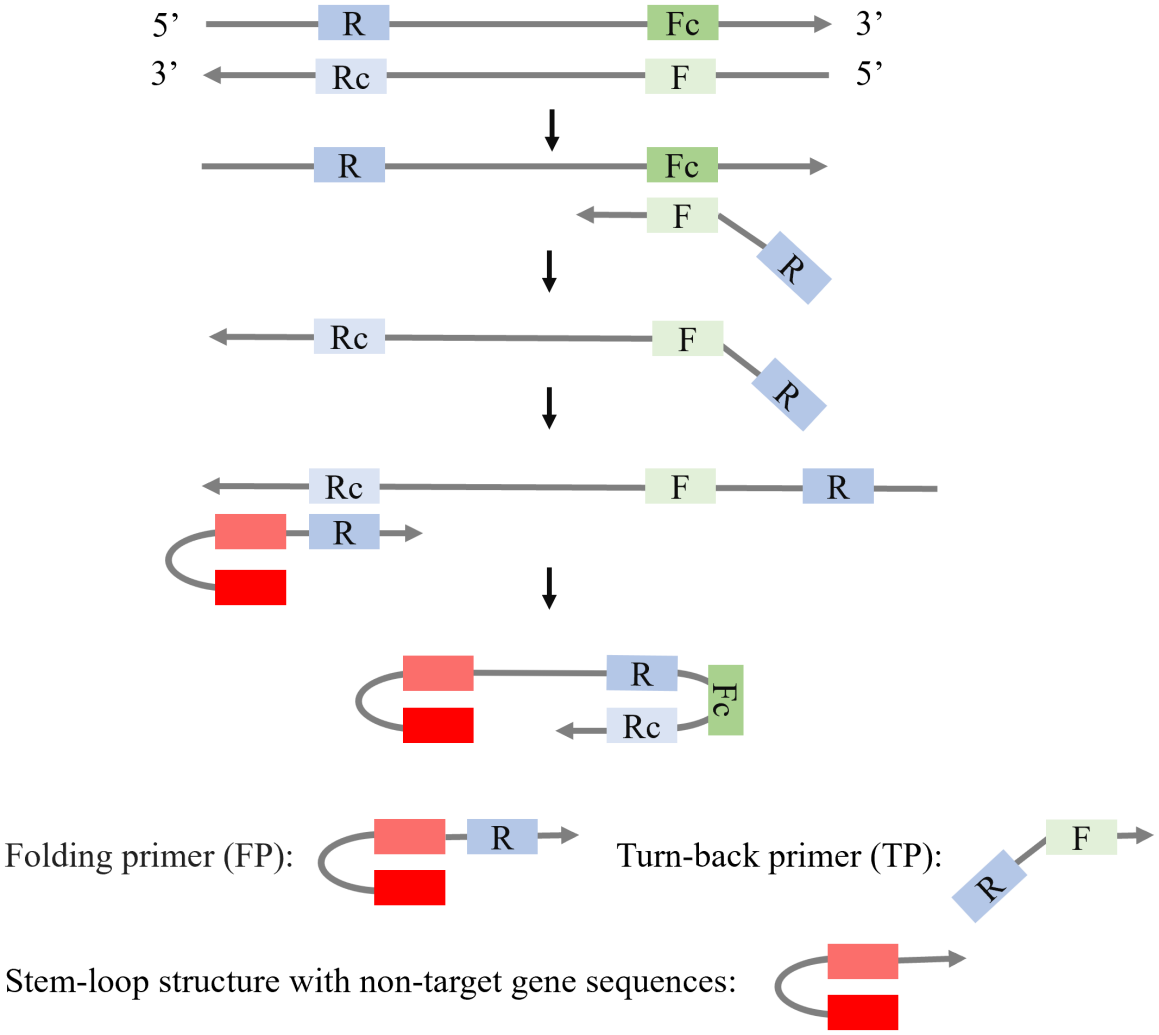
Schematic presentation of the mechanism of LAMP with a Stem-loop primer and a Liner primer.

Besides SLPs, the combination of a stem-loop primer and a linear primer is also applied to isothermal amplification (Figure 4). Mao et al. (2022) modified the stem-loop primers by creating an asymmetric Stem-loop-mediated Isothermal Amplification (ASLAMP). In ASLAMP, PCR primers (F, R) are utilized to modify primers FP and TP. FP forms a stem-loop structure by incorporating two non-target complementary sequences at the 5′ end of R. In TP, R is added to the 5′ end of F. The F sequence in TP recognizes the target gene, resulting in the formation of a complementary single strand containing R and Rc sequences. FP then utilizes this strand as a template to synthesize a new DNA chain, leading to the formation of an asymmetric dumbbell structure that initiates subsequent LAMP. ASLAMP demonstrates a sensitivity and specificity of 100% in the detection of *Shigella*.

## 5 Combination of LAMP with other techniques

### 5.1 Loop-mediated isothermal amplification with RCA

The LAMP technique is commonly utilized for pathogen detection, however, the application for targeting specific RNA sites has not been widely reported. This can be solved by combining with other RNA isothermal amplification methods. Rolling circle amplification (RCA) is an isothermal amplification method that is frequently used for RNA sequence detection (Takahashi et al., 2018). RCA employs circular DNA as a template to generate single-stranded DNA or RNA, which is facilitated by DNA or RNA polymerases. Tian et al. (2019) developed an RCA-actuated LAMP technique utilizing padlock probes, which effectively detected a 10 aM concentration of microRNA. Padlock probes are composed of hybridization regions at the 5′ and 3′ ends for the target sequence, as well as three functional areas (SHS, F1c, F2c). Upon hybridization with the target sequence, the probe forms a circular ssDNA through the action of ligase, initiating RCA and producing ssDNA containing numerous long tandem sequences (F1 + F2 + SHS) with stem-loop structures (F1/F1c and F2) at the 5′ ends. SLPs, which include another stem-loop structure (B1/B1c and B2) and a 3′ terminal ssDNA, can bind to multiple SHS (SLP hybridization sites) regions to create various dumb-bell DNA chains with different stem lengths, thereby initiating LAMP.

Afterward, Gawande et al. (2022) employed the RCA-LAMP technique for detecting cotton leaf curl virus (CLCuV), which overcame the challenge of diagnostic failure in PCR or LAMP assays caused by low virus titers in infected leaves. In addition, Li et al. (2023) developed a method for detecting N6-methyladenosine (m6A) using RCA and LAMP. When padlock probes hybridize with potential m6A sites on targets, they are circularized by DNA ligase to facilitate the LAMP in the absence of m6A modification. However, the presence of m6A modification inhibits the sealing of padlock probes. This method allows for the quantitative detection of m6A targets at specific sites, with a sensitivity as low as 100 aM. The integration of LAMP and RCA methodologies offers a promising approach for the highly sensitive detection of genetic biomarkers and samples with minimal virus titers.

### 5.2 Loop-mediated isothermal amplification with RPA

Among the various isothermal amplification methods, recombinase polymerase amplification (RPA) stands out as a highly sensitive and selective technique that has been utilized for the amplification of a diverse range of target genes (Lobato and O’Sullivan, 2018). A novel approach developed by Chen et al. (2020) combines LAMP with RPA, resulting in a technique known as recombinase-assisted loop-mediated isothermal DNA amplification (RALA). RALA employs the recombinase from *Thermus thermophilus* (TthRecA) in conjunction with a pair of internal primers (FIP, BIP) to facilitate the opening of target double-stranded DNA and initiate LAMP. The strand displacement activity of TthReCA is retained at 60°C, with the recombinase-FIP complex facilitating the opening of double-stranded DNA and guiding the synthesis of a complementary strand to form a new double-stranded DNA. This newly formed DNA is then recognized and opened by the recombinase-BIP complex, leading to the displacement of a single-stranded DNA by the extended BIP. The resulting dumbbell DNA strand initiates LAMP. Wei et al. (2022) developed a real-time fluorescence identification system utilizing RALA for the detection of *Bulbus Fritillariae*, capable of identifying as little as 0.1% of genuine targets in the mixed samples.

In addition to the development of the RALA method, Song et al. (2017) introduced a novel two-stage multiplex isothermal amplification technique known as Rapid Amplification with Multiplex Primer (RAMP). The initial stage of RAMP involves conducting a rapid amplification of RPA at approximately 37°C using external LAMP primers for all targets, followed by a subsequent amplification step in secondary-stage reactors containing internal primers and loop primers specific to each target. This two-stage process allows the LAMP reaction to be completed within 30 min. RAMP can detect 16 pathogens simultaneously, with a sensitivity that surpasses both LAMP and RPA methods by a factor of 10, and a specificity of 100%. In response to the urgent requirement for rapid detection of the SARS-CoV-2 virus, Song (Song et al., 2021) subsequently developed the Peen-RAMP technique. This approach entails initiating RPA within the tube cap, followed by iterative inversion of the reaction tube to facilitate reactant mixing, and culminating in the execution of LAMP within the tube. The Peen-LAMP method has demonstrated the capability to detect 5 copies/reaction of the SARS-CoV-2 virus.

### 5.3 Loop-mediated isothermal amplification with HCR

The utilization of one-step strand displacement (OSD) in combination with LAMP has been demonstrated to effectively differentiate specific and non-specific LAMP amplicons. Du et al. (2015) integrated LAMP with OSD to convert Middle East respiratory syndrome coronavirus and Zaire Ebola virus template information into glucose signals. However, the inability of OSD to amplify the signal results in limited signal production and subsequent application of the LAMP-OSD system. Consequently, the substitution of OSD with hybridization chain reaction (HCR) can address the need for signal amplification.

The HCR, originally developed by the Pierce group, integrates multiple OSD processes to serve as a signal amplifier with high amplification efficiency (Dirks and Pierce, 2004). Dong et al. (2019) further enhanced the versatility of gene diagnostic strategies by combining LAMP with HCR (LAMP-HCR). The signals of LAMP-HCR can be conveniently analyzed using a flow cytometer and a personal glucometer. Various structures including MB Bio-H1, FAM-H1, and FAM-H2 were developed in this research. The MB Bio-H1 comprises a magnetic bead (MB) conjugated with a biotin-labeled H1, where H1 is a hairpin oligonucleotide containing a sequence that is complementary to the Bc-loop and a cyclic single-stranded nucleotide. H2 is the reverse complement of H1. Following LAMP, the hairpin structure of MB Bio-H1 was unfolded through hybridization with the Bc-loop, leading to two OSD reactions. Subsequent hybridization of FAM-H2 with the cyclic single-stranded nucleotide of MB Bio-H1, followed by the binding of FAM-H1 to FAM-H2, triggers the HCR that generates long, nicked dsDNA. The HCR induced by LAMP may lead to the aggregation of a substantial quantity of fluorophores on MBs, which can subsequently be detected through fluorescence signal analysis. Utilizing this method, LAMP-HCR has demonstrated the ability to detect as few as 30 copies of the Norovirus gene in 2% fecal samples.

### 5.4 Loop-mediated isothermal amplification with CRISPR-Cas

The CRISPR-Cas system, consisting of Clustered Regularly Interspaced Short Palindromic Repeats (CRISPR) and associated proteins (Cas), functions as an adaptive immune system in prokaryotes, protecting against the invasion of foreign genetic material. The formation of single guide RNA (sgRNA) occurs through base pairing between trans-activated RNA (tracrRNA) and CRISPR RNA (crRNA) targeting foreign genes. Upon binding to the target, sgRNA activates the Cas protein, inducing nuclease activity for target cleavage (cis-cleavage), thus serving a defensive function (Atçeken et al., 2022; Bhatt et al., 2022). Cas12, Cas13, and Cas14 proteins exhibit both cis-cleavage activity and non-specific nucleic acid sequence cleavage activity (collateral cleavage), which can be harnessed for amplifying fluorescence signals and are commonly used in molecular diagnostics (Harrington et al., 2018; Zavvar et al., 2022).

Li et al. (2019) pioneered the development of a one-step detection system known as HOLMESv2, which utilizes Cas12b and LAMP. Upon binding with the target gene, the Cas12b-sgRNA complex initiates the collateral cleavage activity of Cas12b. This process involves simultaneously cleaving the target dsDNA and the ssDNA fluorescent probes attached to the target, resulting in the release of a fluorescent detection signal. This system can selectively detect single nucleotide polymorphisms (SNPs) and a variety of targets such as viral RNA, human cell mRNA, and circular RNA. Ortiz-Cartagena et al. (2023) developed a precise detection technique utilizing LAMP and CRISPR-Cas13a, effectively identifying OXA-48 and GES Carbapenemases in various clinical specimens, with sensitivity and specificity levels reaching 100%. Wang Q. et al. (2024) introduced a Cas14a1-enhanced LAMP assay in conjunction with Rapid Extraction of Bacterial Genomic DNA, achieving the LOD of 10 aM target genomic DNA. The integration of CRISPR-Cas technology with LAMP has the potential to enhance the sensitivity and specificity of target detection and signal amplification, thereby advancing the development of novel POCT technologies.

### 5.5 Loop-mediated isothermal amplification with biosensors

Biosensors are small devices composed of a biorecognition element, a transducer, and a signal amplifier. The biorecognition element identifies the analytes and generates information, which the transducer then converts into processable signals such as sound, light, or electricity. The signal amplifier enhances and outputs these signals for qualitative or quantitative analysis. Equipped with biological receptors, biosensors are known for their ease of use, portability, and ability to provide real-time analysis. Biosensors utilizing LAMP technology have been extensively researched and applied in POCT. Detailed examples are listed in Table 1.

**Table 1:** LAMP with biosensors.

Technique	Pathogen	Signal transduction material	Readout method	Time	Limit of detection (LOD)	Reference
Electrochemical sensor	*Listeria monocytogenes*	Methylene blue	Cyclic voltammetry (CV)	50 min	1.25 pg/reaction	Rivas-Macho et al. (2023b)
	*Vibrio parahaemolyticus*	Hoechst-33258	Differential pulse voltammetry (DPV)	45 min	0.3 CFU/25 g raw seafood	Kampeera et al. (2019)
	microRNAs (hsa-miR-16-5p, hsa-miR- 191-5p, hsa-miR-423–5p)	Ruthenium hexamine	Linear sweep voltammetry (LSV)	120 min	10^3^–10^6^ copies/50 μL	Rivas-Macho et al. (2023a)
Based on lateral flow sensor	*Plasmodium* spp. *P. knowlesi* *P. vivax* *P. falciparum*	AuNPs	Visual	60 min	Not mentioned	Lai et al. (2023)
	SARS-CoV-2	hCG-probe		120 min	0.5 copies/μL	Yang et al. (2021)
Optical sensor	SARS-COV-2	Au-Ag alloy nanoshells	SPR	75 min	10 copies/ reaction	Ye et al. (2022)
	*Salmonella enterica*	AuNP-Cy5/DNA	SERS	40 min	66 CFU/mL	Xiao et al. (2024)

#### 5.5.1 Electrochemical sensors

Electrochemical sensors detect the analytes using current or electron transfer resistance generated by redox species. A study by Rivas-Macho et al. (2023b) innovated a 3D-printed sensor utilizing LAMP and electrochemical transduction, incorporating methylene blue as the redox-active molecule. The incorporation of methylene blue into the target sequence led to a decrease in the methylene blue reduction current, facilitating detection. This sensor exhibited complete specificity in identifying 12 serotypes of *Listeria monocytogenes*, achieving an LOD of 1.25 pg/reaction. In addition, disposable electrochemical sensors combining LAMP and screen-printed graphene electrodes were developed by Kampeera et al. (2019). Hoechst-33258 is a redox substance designed as that can selectively bind to LAMP amplicons. After the LAMP reaction, the electro-oxidation of Hoechst 33258 will be rapidly quenched, resulting in the reduction of electrochemical current. The method can specially detect *Vibrio parahaemolyticus* within 45 min at the LOD of 0.3 CFU/25 g of raw seafood. Rivas-Macho et al. developed an electrochemical DNA chip based on LAMP for detecting mature microRNAs. The coprecipitation of ruthenium hexamine with coproduced pyrophosphate according to the LAMP reaction causes an increase in the redox current by voltammetry. The chip can detect five kinds of microRNAs in the serum of patients with breast cancer within 2 h (Rivas-Macho et al., 2023a).

#### 5.5.2 Based on lateral flow sensors

The lateral flow assay (LFA) is a simple, cheap, user-friendly method based on an immunochromatographic technique without any special equipment for analysis, making it one of the most popular techniques for POCT. Lai et al. (2023) developed a LAMP-Lateral Flow (LAMP-LF) test strip, which includes various components such as a conjugate pad with streptavidin-bound gold nanoparticles, test lines containing anti-fluorescein isothiocyanate, anti-digoxigenin, anti-cyanine5, anti-dinitrophenol, and a control line with biotinylated-BSA. The detection of four targets, including *Plasmodium* spp.-LAMP product, *P. falciparum*-LAMP product, *P. vivax*-LAMP product, and *P. knowlesi*-LAMP product, is achieved through antigen–antibody reactions. The LAMP-LF test has demonstrated a sensitivity of 100% and a specificity of 97.8% in their study. Yang et al. reported a portable microfluidic platform integrating LAMP and pregnancy test strips for the POCT of SARS-CoV-2. The researchers designed human chorionic gonadotropin probes that hybridized with the specific loop region of LAMP amplicons. Probes are only present on the control line of pregnancy test strips. The detection is completed within 120 min with an LOD of 0.5 copies/uL (Yang et al., 2021).

#### 5.5.3 Optical sensors

Surface plasmon resonance (SPR) sensors are optical sensors that detect the analytes on a surface by measuring changes in the refractive index in real-time. The binding event between the analytes and biorecognition molecules on the sensor induces substrate surface change, which is transduced to a change in the angle. Ye et al. developed plasmonic sensors based on gold-silver (Au-Ag) alloy nanoshells called Plasmonic-LAMP. During the reaction, LAMP amplicons are digested by restriction enzymes to form short repeats, which can be denatured into oligonucleotides for subsequent DNA hybridization linked with plasmonic sensors. In the SARS-CoV-2 RNA detection, plasmonic-LAMP achieves an LOD of 10 copies/reaction. The contamination from non-template amplification is further eliminated (Ye et al., 2022). Xiao et al. developed an integrated assay that combines LMAP and SERS by using specifically designed Raman active gold nanoparticles (AuNPs). The AuNPs modified with Cy5-DNA specifically bind to the complementary sequence of target DNA, and then form a double-stranded DNA structure to protect the Cy5-DNAs complex from nuclease digestion. On the contrary, in the absence of target DNA, the S1 nuclease digests the DNA modified on the surface of AuNPs, triggering the release of Cy5, which then induces a significant reduction in Raman signal intensity. The LAMP-SERS assay can successfully detect *Salmonella enteritidis* in milk samples, and its LOD is 66 CFU/mL (Xiao et al., 2024).

### 5.6 Loop-mediated isothermal amplification with microfluidic technology

Microfluidic technology has the potential to streamline sample pre-processing and amplification product detection processes, presenting a wide range of potential applications. Furthermore, microfluidic technology allows for the utilization of various closed reaction chambers of different shapes for LAMP, effectively preventing aerosol contamination and enabling the detection of multiple products simultaneously. Jin et al. (2021) have successfully incorporated LAMP into a highly automated dual-sample microfluidic chip. This innovative chip has the capability to conduct 22 genetic analyses from two samples within a time frame of 35 min, while concurrently identifying 10 different waterborne pathogens. The chip’s detection limit spans from 7.92 × 10^-3^ to 9.54 × 10^-1^ pg of genomic DNA from pure bacteria per reaction, with a sensitivity of 93.1% and a specificity of 98.0%. Jiang et al. (2023) introduced a novel approach known as single-molecule RNA capture-assisted digital RT-LAMP (SCADL), which combines droplet generation, RT-LAMP, and result analysis. In SCADL, MBs are functionalized with oligonucleotide probes to facilitate the capture of target sequences by capture probes that bind to oligonucleotide probes. The resulting complex of target sequences and MBs, along with the RT-LAMP reaction mixture, is partitioned into more than 2 × 10^4^ droplets for subsequent quantitative analysis based on the fluorescent signals within the droplets. SCADL is capable of detecting the SARS-CoV-2 N gene in under an hour, with an LOD of 10 copies/μL.

Compared to microfluidic chips, capillary arrays are more cost-effective and easier to use. Li et al. (2020) introduced a mini-disk capillary array combined with LAMP for multiplexing nucleic acid detection. The capillary array features a hydrophilic inner surface and a hydrophobic outer surface, with fixed primers on the inner surface. The LAMP reaction mixture located in the central orifice of the disk is dispersed to individual capillaries via capillary action to facilitate the reaction process. The method has the capability to identify five genetically modified elements with an LOD of 25 copies/reaction. Zhu et al. (2023) developed a high-throughput detection platform using a Hive-Chip capillary array and RT-LAMP technology, operated via an iPad interface. This platform enables the simultaneous testing of 48 samples and the identification of seven genes specific to SARS-CoV-2.

## 6 The applications of LAMP

As discussed earlier, LAMP technology can be used on its own or in combination with other technologies to detect a variety of pathogens. To further illustrate the significance of LAMP in nucleic acid detection, additional examples of its applications in food safety, animal and plant pathogens, and human diseases are discussed. Detailed applications are summarized in Table 2.

**Table 2:** The applications of LAMP.

Application	Target	Detection method	Limit of detection (LOD)	Reference method	Sensitivity	Specificity	Sample	Time	Reference
Food safety	P-CaMV35S P-FMV35S pat NPT II ADH1	Colorimetric detection	25 copies/reaction	PCR	Not mentioned	Not mentioned	GM maize	40 min	Li et al. (2020)
	*Campylobacter jejuni*	Turbidity and UV fluorescence test	10^3^ CFU/mL	Microbe culture	96.9%	95.8%	Chicken	180 min	Jainonthee et al. (2022)
	*Echinostomatidae metacercaria*	Lateral flow dipstick	50 pg/mL	Microscopic examination	84.62%	100%	Edible snails	70 min	Panich et al. (2024)
	Dairy	Lateral flow strip	1% content	RT-PCR	100%	100%	Commercially available samples	60 min	Wang et al. (2024a)
Animal and plant pathogens	*Phytophthora ramorum*	Colorimetric detection	30–0.03 ng/μL	RT-PCR	Not mentioned	Not mentioned	Oak	60 min	Søndreli et al. (2023)
	Avian Influenza Virus	Colorimetric detection with Evagreen	10^0.8^ EID50 /reaction	RT-PCR	Not mentioned	Not mentioned	Birds oropharyngeal and cloacal swabs	30 min	Golabi et al. (2021)
	Decapod iridescent virus 1 (DIV1)	Microfluidic chip	10 copies/reaction	qPCR	Not mentioned	100%	*Penaeus vannamei*	40 min	Hu et al. (2023)
	White spot syndrome virus (WSSV)		10^2^ copies/reaction						
	*Enterocytozoon hepatopenae*i (EHP)		10 copies/reaction						
Human diseases	*Plasmodium knowlesi* *P. vivax* *P. falciparum*	Lateral flow assay	Not mentioned	Microscopy and PCR	100%	97.8%	Blood samples	60 min	Lai et al. (2023)
	Human enterovirus 71(EVA71)	Fluorescence detection	11.2 copies/ 25 μL	RT-qPCR	100%	83.3%	Throat swabs and rectal swabs	30 min	Zhang et al. (2024)
	Coxsackievirus A16 (CVA16)		49.6 copies/ 25 μL						
	Coxsackievirus A6 (CVA6)		11.4 copies/ 25 μL						
	Coxsackievirus A10 (CVA10)		20.5 copies/ 25 μL						
	Severe fever with thrombocytopenia syndrome virus (SFTSV)	Fluorescence detection	Not mentioned	RT-qPCR	Not mentioned	Not mentioned	Plasma samples	60 min	Tian et al. (2023)
	*Mycobacterium tuberculosis* (MTB)	Lateral flow assay	100 fg/reaction	RT-PCR	100%	98.04%	Sore throat wipes	80 min	Yang et al. (2023)

### 6.1 Food safety

Food safety is a persistent and significant challenge that greatly impacts human life. In recent years, LAMP technology has played a crucial role in detecting food-borne pathogens, genetically modified organisms, and counterfeit foods. To detect adulterated dairy products, Wang et al. developed a LAMP-based microfluidic system capable of detecting dairy components with concentrations as low as 1% within 60 min. This platform was validated by 35 commercially available samples, which demonstrated 100% specificity and accuracy, compared to real-time PCR (Wang N. et al., 2024). Panich et al. utilized a visual LAMP coupled with a lateral flow dipstick assay to detect *Echinostomatidae metacercaria* in edible snail samples. By detecting 110 edible snail samples and comparing them with microscopic examination, the method showed a clinical sensitivity of 84.62% and a specificity of 100%, with an LOD of 50 ug/uL (Panich et al., 2024). *Campylobacter jejuni*, common in poultry, is a leading cause of food-borne illnesses worldwide. Jainonthee et al. developed a LAMP detection method with sensitivity and specificity of 96.9 and 95.8%, respectively. This method enables the detection of *Campylobacter jejuni* in chicken samples within 3 h, with an LOD of 10^3^ CFU/mL (Jainonthee et al., 2022).

### 6.2 Animal and plant pathogens

Detecting pathogens in animals and plants is essential for disease prevention and minimizing industry losses. *Phytophthora ramorum*, an invasive oomycete responsible for Sudden Oak Death, poses a significant forestry threat worldwide. Søndreli et al. developed a species-specific LAMP to distinguish among the four lineages of *Phytophthora ramorum*, that correctly identifying 190 samples from more than 200 field samples for pedigree determination (Søndreli et al., 2023). Avian influenza virus outbreaks cause significant economic losses in the poultry industry. Golabi et al. designed a real-time reverse transcription LAMP to detect 9 different subtypes of avian influenza virus. The method detects the virus in complex samples using straightforward heat treatment steps, eliminating the need for RNA extraction. It matches the sensitivity of the gold standard RT-PCR, with an LOD of 10^0.8^ EID50/reaction (Golabi et al., 2021). Decapod iridescent virus 1, White spot syndrome virus, and *Enterocytozoon hepatopenaei* pose serious threats to the global shrimp farming industry. Hu et al. developed a LAMP-based microfluidic chip detection system, which demonstrated 100% specificity and good stability compared to a typical qPCR assay (Hu et al., 2023).

### 6.3 Human diseases

In recent years, LAMP has been widely used for rapid detection of pathogenic bacteria. Hand, foot, and mouth disease (HFMD), caused by various enteroviruses, is a major global public health concern. Zhang et al. developed a multiplex high-fidelity DNA polymerase LAMP assay that detects Human enterovirus 71, Coxsackievirus A16, Coxsackievirus A6, and Coxsackievirus A10 in clinical samples within 30 min, with 100% sensitivity and specificity (Zhang et al., 2024). Severe fever with thrombocytopenia syndrome (SFTS), an acute infectious disease prevalent in East Asia, can be effectively diagnosed using LAMP, especially in resource-limited regions. Tian et al. evaluated the on-site testing ability of LAMP with 279 plasma samples from SFTS patients, revealing a sensitivity of 81.9% and a specificity of 96.3% (Tian et al., 2023). Tuberculosis, caused by *Mycobacterium tuberculosis*, is a chronic infectious disease. Yang et al. developed a simple and low-cost detection sensor combining LAMP and lateral flow immunoassay technology, which completes detection within 80 min and has an LOD of 100 fg/reaction (Yang et al., 2023).

## 7 Conclusion

LAMP technology has undergone significant developments and applied to various fields. Since its first introduction in 2000 by Notomi, LAMP has been greatly improved, particularly in primer design and polymerase engineering, which solidifies its status as a potent method for rapid nucleic acid detection. The integration of LAMP with other techniques such as RCA, RPA, CRISPR-Cas systems, biosensors, and microfluidic technology has further expanded its utility, enabling sensitive and specific detection of various targets including pathogens, genetic markers, and biomolecules. The continuous improvement of LAMP technology is making detection devices smaller and more portable, such as personal glucometers, smartphones, flow cytometers, etc., making them more suitable for POCT, especially in areas with limited medical resources. These synergistic applications have paved the way for the development of novel diagnostic platforms with enhanced sensitivity, specificity, and portability. Moving forward, continued research and innovation in LAMP technology hold promise for addressing emerging challenges in healthcare, agriculture, environmental monitoring, and beyond, ultimately contributing to improved disease diagnosis, surveillance, and management on a global scale.

## Author contributions

NY: Data curation, Investigation, Resources, Writing – original draft, Writing – review & editing. HZ: Writing – original draft. XH: Writing – review & editing. ZL: Writing – review & editing. YL: Writing – review & editing.
